# Hybridization in an isolated population of blesbok and red hartebeest

**DOI:** 10.1002/ece3.11194

**Published:** 2024-04-01

**Authors:** Anna M. van Wyk, Erika Schulze, Kim Labuschagne, Seeng Thamae, Antoinette Kotzé, Desiré Lee Dalton

**Affiliations:** ^1^ South African National Biodiversity Institute Pretoria South Africa; ^2^ Molecular Ecology and Evolution Program (MEEP), Department of Biochemistry, Genetics and Microbiology University of Pretoria Pretoria South Africa; ^3^ Department of Economic, Small Business Development Tourism and Environmental Affairs Bloemfontein South Africa; ^4^ Department of Genetics University of the Free State Bloemfontein South Africa; ^5^ School of Health and Life Science Teesside University Middlesbrough UK

**Keywords:** blesbok, hybridization, microsatellites, red hartebeest, sterile offspring

## Abstract

Hybridization in antelope species has been widely reported in South African national parks and provincial reserves as well as on private land due to anthropogenic effects. In a closed management setting, hybridization may occur due to the crossbreeding of closely related species with unequal sex ratios, resulting in either sterile or fertile offspring. In this study, we used molecular techniques to evaluate the risk of anthropogenic hybridization between blesbok (*Damaliscus pygargus phillipsi*) and red hartebeest (*Alcelaphus buselaphus caama*) in an isolated group that purposely included the two species with unequal sex ratios (one red hartebeest male and 19 male and female blesbok). Genetic analysis based on microsatellites confirmed the presence of seven hybrid individuals. Mitochondrial analysis verified that hybridization occurred between blesbok females and the red hartebeest male. STRUCTURE and NEWHYBRIDS classified the hybrids as F1. It is suspected that the hybrid individuals were sterile as the males had undeveloped testes and only F1 hybrids were detected. Thus, the risk of hybridization between these two species may be limited in the wild. In captive settings, genetic monitoring should be included in management plans for blesbok and red hartebeest to ensure that the long‐term consequences of wasted reproductive effort are limited.

## Graphical abstract

A genetic investigation of hybridization between blesbok and red hartebeest determined that offspring are sterile. These results support that the risk of hybridization between blesbok and red hartebeest may be limited in the wild and would result in wasted reproductive effort.

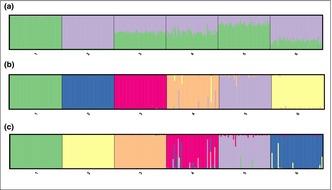

## 1 INTRODUCTION

South Africa, as a signatory to the Convention on Biological Diversity (CBD) has implemented legislative measures for environmental protection. Establishment of national parks, game reserves and other protected areas of natural habitat is one of the principle strategies used to conserve unique biodiversity. Approximately 9% of the countries land has been identified as protected areas. However, the private sector also plays a major role in the protection and management of antelopes. It is estimated that the private wildlife industry is 2.2 times greater than the state protected area network of the country (Els, 2017). Privately owned wildlife are used for recreational hunting, trophy hunting, wildlife meat production, breeding of wildlife and ecotourism (game viewing, walking safaris and photographic safaris) (Du Plessis, 1997). Antelopes in protected areas and on private land, face several threats including habitat degradation and destruction, disease, small population size and lastly hybridization.

Natural hybridization refers to the interbreeding of individuals from two genetically distinct species or populations (Arnold, 1997). This natural process is considered rare and may have substantial evolutionary significance, increasing adaptive capacity and species diversity (Abbott et al., 2013). However, others consider natural hybridization to be maladaptive due to reduced frequency and fitness of hybrids in comparison to parental genotypes (Moore & Price, 1993). In contrast, anthropogenic activities such as introduction of plants or animal (eg. translocation), habitat modification and/or fragmentation has dramatically increased the rates of hybridization worldwide (Stronen & Paquet, 2013). Antropogenic hybridization is reported to have resulted in the extinction of several taxa and it has been suggested that conservation policies that are designed to reduce anthropogenic hybridization should be adopted (Allendorf et al., 2001). Hybridization between species or sub‐species can have a variety of outcomes, including hybridization with introgression (Rhymer & Simberloff, 1996) where offspring are fertile. In these cases, there is a risk of extinction via introgression resulting in complete admixture (Allendorf et al., 2001; Rhymer et al., 1994; Rhymer & Simberloff, 1996). Hybridization without introgression can occur where hybrid offspring are sterile. Here, populations may decline due to wasted reproductive effort (Allendorf et al., 2001). However, the long‐term consequences of wasted reproductive effort in small isolated populations are currently unknown. Hybridization is considered a real threat in South Africa as wildlife species are extensively translocated outside of their historic distribution ranges onto private land as part of wildlife management and commercial breeding (Spear & Chown, 2009). In closed management settings, multiple species may occur on the same property where there are few or no conspecific mates (Dalton et al., 2014; Grobler et al., 2011) which may contribute to an increased risk of hybridization. In addition, hybridization can occur due to loss of reproductive barriers between previously isolated evolutionary lineages (Green & Rothstein, 1998).

Red hartebeest (*Alcelaphus buselaphus caama*) is suspected to hybridise with blesbok (*Damaliscus pygargus phillipsi*), bontebok (*D. p. pygargus*) or tsessebe (*D. lunatus lunatus*) on private land (Venter & Child, 2016). Hybridization between red hartebeest and blesbok has been previously confirmed based on cytogenetic analyses (Robinson et al., 1991). The authors identified F1 hybrids with the number of chromosome being intermediate (2*n* = 39) in comparison to parental species (red hartebeest 2*n* = 40 and blesbok, 2*n* = 38). One of the hybrid males was further reported as sterile based on azoospermia and lack of germ cells in seminiferous tubule cross‐sections (Robinson et al., 1991). The blesbok is an abundant antelope species that is listed as least concern on the International Union for Conservation of Nature (IUCN) Red List of Threatened Species (Lloyd & David, 2008). Blesbok were historically distributed across the Highveld grasslands of the Free State, Gauteng, north‐western KwaZulu‐Natal and parts of the Karoo in the Eastern and Northern Cape provinces of South Africa (Skinner & Chimimba, 2005). Red hartebeest occurs in dry, arid regions of Namibia, the Kalahari, southern Botswana, and is widespread in South Africa, with the exception of the Lowveld of Mpumalanga and Limpopo and Northern Kwazulu‐Natal. The species is listed as least concern (IUCN SSC Antelope Specialist Group, 2017; Venter & Child, 2016). Currently, both species are found on private land and in national parks and provincial reserves within and outside of their natural distribution range (Power, 2014). In South Africa, the total number of mature red hartebeest and blesbok were estimated at 38,511 (2013–2014 counts) and 54,426 (2010–2016 counts) respectively with 14,849 red hartebeest and 17,235 blesbok found in protected areas across the country (Dalton et al., 2017; Venter & Child, 2016).

Thus far, identification of hybridization between the red hartebeest and blesbok based on genetic markers has not been reported. Molecular analysis has been identified as an accurate method to identify hybrids and detect introgression between taxa (Avise & Hamrick, 1996). Various nuclear and uniparental molecular markers can be used including; mitochondrial DNA (mtDNA; Abbott et al., 2013), Y‐chromosome markers (Petit et al., 2002), microsatellites (Haasl & Payseur, 2011) and single nucleotide polymorphisms (SNPs) (Herrero‐Medrano et al., 2013). Identification of anthropogenic hybridization based on genetic markers has been reported in a number of antelope species in South Africa including the blue (*Connochaetes taurinus*) and black wildebeest (*C. gnu*; Grobler et al., 2011, 2018), bontebok and blesbok (van Wyk et al., 2013) nyala (*Tragelaphus angasii*) and kudu (*T. strepsiceros*; Dalton et al., 2014). In this study, we used nuclear and mtDNA loci to detect hybridization in an isolated population established in 2013 where blesbok and red hartebeest were deliberately mixed with unequal sex ratios. Here, the aim was to determine if hybridization would occur between these two species and to determine the extent of hybridization. In addition, we explore the potential for hybrid fertility, in order to assess the threat that this phenomenon currently poses to both species.

## 2 MATERIALS AND METHODS

### 2.1 Study site and sample collection

Rustfontein Dam Nature Reserve (29°16′15″ S, 26°37′1″ E) is situated 10 km south of the N8 national road between Bloemfontein and Thaba Nchu in the Free State Province, South Africa. The dam provides water for domestic, industrial and irrigation purposes. The founder group, introduced in 2008, consisted of five blesbok. One adult red hartebeest male was released into the herd in June 2013 and 19 blesbok were added from an adjacent reserve in July 2013 (sex of blesbok was not recorded). The mixing of two species with unequal sex ratios is a common occurance in South Africa on private land and in this study was done deliberately in order to determine if hybridization would occur. In 2018, the entire group was culled and tissue samples were taken from the remaining 26 individuals. Futher details of the experimental population per year is indicated in Figure S1. In addition, reference samples of red hartebeest (*n* = 13) and blesbok (*n* = 20) were obtained from the South African National Biodiversity Institute (SANBI) Biobank. The reference samples are part of the Biobank collection and represent various localities and populations within the current distribution ranges of both species.

### 2.2 Genetic hybrid identification

#### 2.2.1 Microsatellite DNA analysis

DNA was extracted from tissue samples using the ZR Genomic DNA™ Tissue MiniPrep (Zymo Research Corporation) following the extraction protocol. Cross‐species autosomal microsatellites markers (*n* = 21, Table S1) developed for bovids (cattle, goat, sheep or blesbok) were used to genotype all individuals (Bhebhe et al., 1994; Bishop et al., 1994; Buchanan et al., 1994; Dalton et al., 2011; Ede et al., 1995; FAO, 2011; Massey & Georges, 1992; Sonstegard et al., 1997; Sunden et al., 1993; Toldo et al., 1993; Vaiman et al., 1992, 1994). Based allelic size range and fluorescent dye of individual primer pairs, sets of primers were combined and tested in multiplexed PCR reactions. Polymerase Chain Reaction (PCR) amplification was conducted in a 12.5 micro litre (μL) reaction volume consisting of Ampliqon Taq DNA polymerase RED (Lasec, Cape Town, SA), 5′‐fluorescent dye labelled forward primer and reverse primer (0.5 micro Molar (μM) each) and 50 nano gram (ng) genomic DNA template. The conditions for PCR amplification were as follows: 5 min at 95°C denaturation, 35 cycles for 30 s at 95°C, 30 s at 50–62°C (refer to Table S1 for annealing temperatures) and 30 s at 72°C, followed by extension at 72°C for 10 min in a T100™ Thermal Cycler (Bio‐Rad Laboratories, Inc. Hercules, CA, USA). PCR products were run against a Genescan™ 500 LIZ™ internal size standard on an ABI 3130 genetic analyser (Applied Biosystems, Inc., Foster City, CA, USA) and were genotyped using GeneMapper® v. 4.0 (Applied Biosystems, Inc., Foster City, CA, USA).

Analysis was performed for red hartebeest, blesbok and putative hybrids separately. In order to detect genotyping errors, allele dropout and null alleles in the reference samples (red hartebeest and blesbok) MICRO‐CHECKER (van Oosterhout et al., 2004) was used. In addition, in order to identify deviations from Hardy–Weinberg (HW) proportions of genotypes, analysis was conducted in Arlequin 3.5 (Excoffier et al., 2005; Excoffier & Lischer, 2010) using the following settings: Markov Chain length of 105 and 100,000 dememorization steps. Lastly, the presense or absence of linkage disequilibrium was verified using exact test described by Guo and Thompson (1992). A total of 100 initial conditions followed by ten permutations, based on the Sequential Bonferroni correction was used to adjust for multiple tests at a significant level of .05 (Rice, 1989). Mean number of alleles per locus (A), effective number of alleles (*N*_e_), observed heterozygosities (*H*_o_) and unbiased heterozygosities (*H*_z_ = expected heterozygosity adjusted for unequal sample sizes; Nei, 1987) were used to estimate the level of genetic diversity (GenAlEx 6.5; Peakall & Smouse, 2006, 2012). In order to assess the level of genetic differentiation between the two species and *F*_ST_‐based hierarchical analysis of molecular variance (AMOVA; Excoffier et al., 1992) was calculated using Arlequin 3.5 (Excoffier et al., 2005; Excoffier & Lischer, 2010).

In order to identify hybrid individuals STRUCTURE 2.3.4 (Falush et al., 2007; Hubisz et al., 2009; Pritchard et al., 2000) and NEWHYBRIDS 1.1 (Anderson & Thompson, 2002) was used. The STRUCTURE analysis was performed using a model that assumes admixture, correlated allele frequencies and without prior population information for ten replicates each from *K* = 1–2, with a run‐length of 700,000 Markov Chain Monte Carlo repetitions, following a burn‐in period of 200,000 iterations. NEWHYBRIDS determines the probability of each individual belonging to one of six categories including the two parental species, F1 and F2 hybrids, and backcrosses to each of the parental species. The NEWHYBRIDS analysis was performed in ten replicates runs for each prior (Jeffreys and Uniform) with 200,000 burn‐in iterations and a total run length of 1,000,000. CLUMPAK (Cluster Markov Packager Across *K*; Kopelman et al. (2015)) was used to graphically represent the runs for both STRUCTURE and NEWHYBRIDS. CLUMPAK obtains the membership coefficient matrices (Q‐matrices) of replicate runs using CLUMPP (Jakobsson & Rosenberg, 2007), employing as a default, the LargeKGreedy algorithm with 2000 random input sequences. CLUMPAK employs DISTRUCT (Rosenberg, 2004) to graphically display the average run representing each cluster. For STRUCTURE, the average proportion of membership (*q*i) of the sampled groups to the inferred clusters was assessed. Individuals were assigned to the inferred clusters using an threshold of *q*i > 0.9 (Barilani et al., 2007). Thus, individuals with *q*i values higher than 0.9 were identified as pure whereas individuals with values lower than 0.9 were classified as hybrid. Vähä and Primmer (2006) indicated that NEWHYBRIDS is less sensitive than STRUCTURE to differentiate between nonadmixed and admixed individuals, therefore a 0.7 probability of an individual belonging to a single category was selected as a threshold value (Costa et al., 2017; Gagnaire et al., 2009; Gunnell et al., 2008; Marie et al., 2011; Uwimana et al., 2012).

In order to maximise the accuracy of assignment, simulated genotypes were created using HYBRIDLAB (Nielsen et al., 2006). The genotypes of reference red hartebeest (*n* = 13) and blesbok (*n* = 21) were used to create 50 of each parental group and in turn to create the simulated hybrid genotypes. A dataset consisting of 300 individuals were produced consisting of 50 genotypes each belonging to red hartebeest, blesbok, F1 hybrids, F2 hybrids, backcross to red hartebeest and backcross to blesbok. The simulated dataset was analysed with STRUCTURE and NEWHYBRIDS as described above.

Pairwise relatedness was calculated between all individuals in the putatitve hybrid group, using the Lynch and Ritland estimator in GenAlEx version 6.5 (Peakall & Smouse, 2006). The relatedness coefficients (*r*) varies from 0 to 1, where .5 indicates that individuals are first‐order relatives (parent and offspring or full‐siblings) and a relatedness coefficient of .25 indicates second‐order relatedness (half‐siblings, grandparents, aunts/uncles or to niece/nephews).

#### 2.2.2 Mitochondrial DNA sequencing

The hybrid individuals identified through STRUCTURE and NEWHYBRIDS were further classified by means of cytochrome b (Cytb) amplification and sequence analyses to confirm maternity. Primers L14724 and H16498 (Hsieh et al., 2001) were used to target a 427 base pair (bp) region of the gene and amplification was conducted in a final reaction volume of 25 μL using DreamTaq Green PCR Master Mix (Thermo Scientific), forward and reverse primers (10 pico mole (pmol) each), and template DNA (20 ng). The cycling conditions were as follows; 2 min at 94°C, 5 cycles for 30 s at 94°C, 40 s at 45°C, 1 min at 72°C, followed by 35 cycles for 30 s at 94°C, 40 s at 51°C, 1 min at 72°C and finally 72°C for 10 min in a T100™ Thermal Cycler. PCR purification was conducted in a final reaction volume of 22.5 μL consisting of FastAP™ Thermosensitive Alkaline Phosphatase (Thermo Fisher Scientific Inc.) (1 U/μL), exonuclease I (20 U/μL) and PCR product (20 μL) in a T100™ Thermal Cycler at the following conditions; 37°C for 15 min followed by 85°C for 15 min. After PCR purification, cycle sequencing was conducted using BigDye® Terminator v3.1 Cycle Sequencing Kit (Life Technologies, USA) in a final reaction volume of 10 μL, including purified PCR product (5 μL) and forward or reverse primer (10 pmol). Cycle sequencing was carried out in a T100™ Thermal Cycler at the following conditions; 94°C for 2 min followed by 40 cycles for 10 s at 85°C, 10 s at 50°C, 2 min 30 s at 60°C. BigDye® XTerminator™ Purification Kit (Applied Biosystems, Inc., Foster City, CA, USA) was used for sequencing clean‐up according to the manufacture's protocol. Analysis of the sequences was carried out on the ABI 3130 genetic analyser. Sequence‐Analysis v. 4.0 (Applied Biosystems, Inc., Foster City, CA, USA) was used to visualise the sequences. Resulting sequence chromatograms were viewed and edited in the Chromas program embedded in MEGA5 (Tamura et al., 2011) prior to performing a BLAST nucleotide search (www.ncbi.nm.nih.gov/blast). Maximum likelihood (ML) analyses were inferred in MEGA5 while the best fit model of sequence evolution was selected under the Akaike Information Criterion (AIC) in jModeltest (Posada, 2008). Nodal support for the Likelihood (ML) tree was assessed through 10,000 non‐parametric bootstrap replications.

## 3 RESULTS

### 3.1 Genetic analysis and assignment testing

Amplification was obtained for all 21 cross‐species autosomal microsatellites markers. However, three markers (SRCRSP8, BB05 and SPS113) were removed from the final data set as amplification was not achieved for more than 30% of the genotypes in the red hartebeest reference population. Null alleles were not detected in the red hartebeest population (Table S2). In addition, seven markers (BB10, BB08, OARCP26, BM2113, BB04, ETH10 and INRA128) in the blesbok population showed null allele frequencies higher than 0.2 at all four algorithms (Oosterhout, Chakraborty, Brookfield 1 and Brookfield 2). Significant deviations from HW equilibrium were observed in one marker (BB10) in the blesbok following Bonferroni corrections. Significant linkage disequilibrium (LD) was only observed in the putative hybrid group which may be a consequence of it being admixed. Markers containing possible null alleles and deviations from HW equilibrium were removed from the final dataset. Thus, the final data set included 11 cross‐species microsatellite.

Genetic diversity for each species and putative hybrids is summarised in Table 1. The genetic diversity in the red hartebeest was higher compared to the blesbok with the putative hybrid individuals being intermediate. Mean number of alleles per locus was 5.80, 2.80 and 4.00 while the average Ne was 3.58, 2.12 and 2.31 for red hartebeest, blesbok and putative hybrid individuals respectively. Observed heterozygosity was 0.55 (red hartebeest), 0.40 (blesbok) and 0.46 (putative hybrids) while H_z_ was 0.56 (red hartebeest), 0.46 (blesbok) and 0.53 (putative hybrids) (Table 1).

**TABLE 1:** Genetic diversity estimates for red hartebeest, blesbok and putative hybrid populations.

Population	Sample size (*N*)	Mean number of alleles per locus (A)	Effective number of alleles (*N*_e_)	Observed heterozygosity (*H*_o_)	Unbiased heterozygosity (*H*_z_)
Red hartebeest	13	5.80	3.58	0.55	0.56
Blesbok	20	2.80	2.12	0.40	0.46
Putative hybrids	26	4.00	2.31	0.46	0.53

Abbreviations: A, Mean number of alleles per locus; *H*_o_, Observed heterozygosity; *H*_z_, Unbiased heterozygosity; *N*, sample size; *N*_e_, effective number of alleles.

**FIGURE 1:**
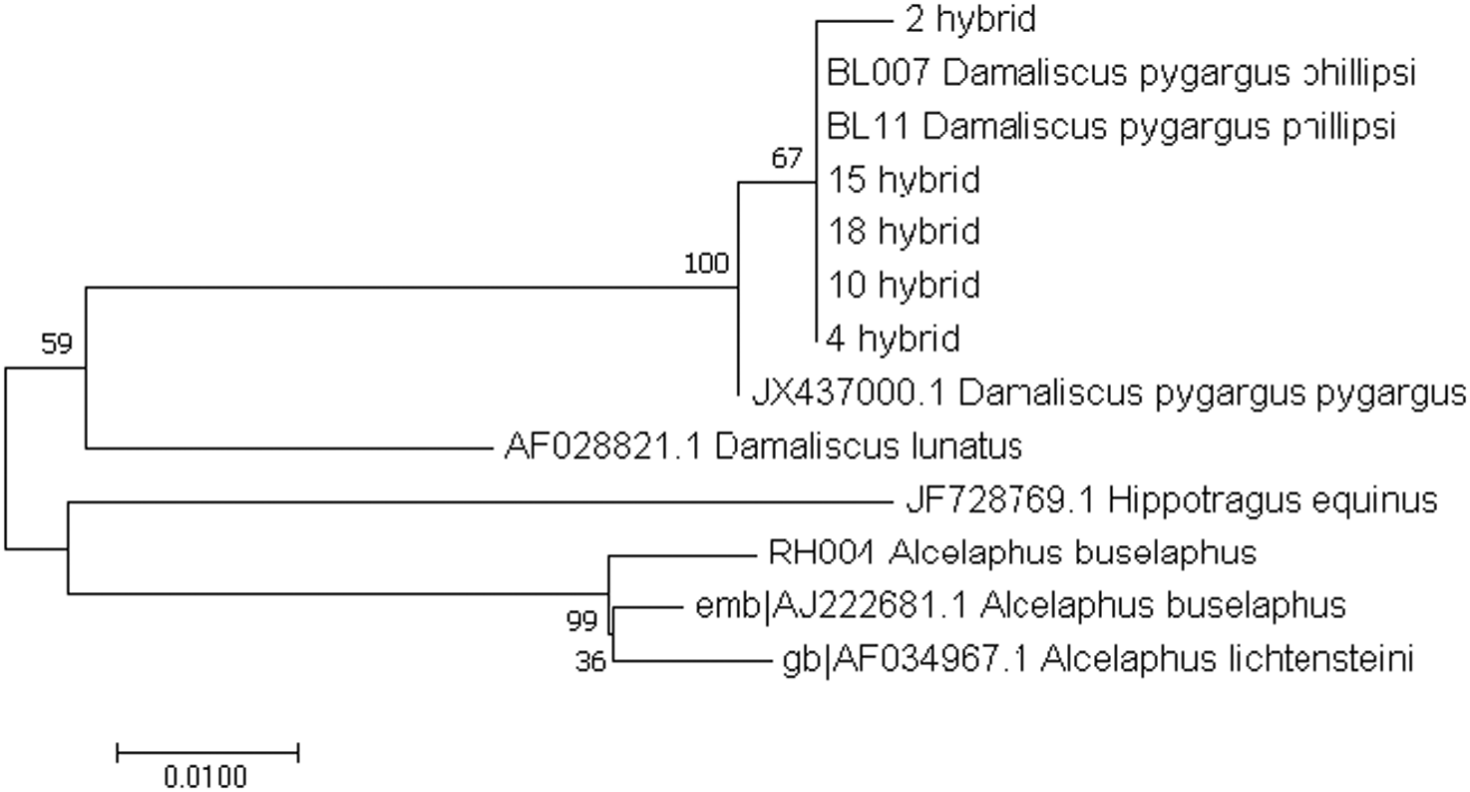
Maximum likelihood (ML) phylogenetic tree of blesbok, red hartebeest and hybrids (001, 002, 004, 010, 015 and 019) based on cytochrome b in combination with reference samples acquired from Genbank. All reference samples are prefixed with relevant Genbank accession numbers, while reference samples (generated in this study) are indicated with BL or RH. ML bootstrap support values given at the nodes. Red hartebeest and blesbok fall into two distinct clades and all hybrid animals have blesbok mitochondrial lineages.

In the reference population, a total of 91 alleles were observed with 23 specific to blesbok, 60 to red hartebeest and eight shared (Table S3). Analysis by AMOVA (45%) and pairwise *F*_ST_ (*F*_ST_ = 0.45286, *p* < .001) indicated a high proportion of genetic differentiation among populations. This was further supported by phylogenetic analysis where distinct clades for blesbok and red hartebeest were recovered with 100% bootstrap support (Figure 1). Hybrid individuals clustered with blesbok indicating that hybridization involved mating of blesbok females with the male red hartebeest.

**FIGURE 2:**
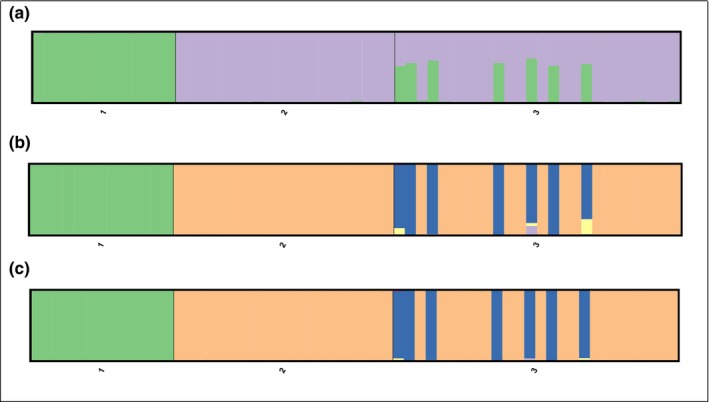
Genetic differentiation analysis between populations based on (a) STRUCTURE analysis (performed with *K* = 2) and NEWHYBRIDS using (b) Jeffreys prior and (c) Uniform prior of 1 = red hartebeest, 2 = blesbok and 3 = putative hybrids.

Both STRUCTURE and NEWHYBRIDS showed similar results, identifying seven F1 hybrids and classifying the rest of the individuals (19) as blesbok (Figure 2; Table S4). In STRUCTURE, the average proportion of membership for both pure populations was *q*_i_ > 0.989. Using the criterion of *q*_i_ > 0.90 suggested by Barilani et al. (2007) to identify individuals as either pure or hybrid, 19 individuals were classified as blesbok with *q*_i_ > 0.983 and seven F1 hybrids were identified with *q*_iblesbok_ ranging from 0.373 to 0.482 (Table 2, Table S4). NEWHYBRIDS indicated similar results for both Jeffreys and Uniform priors using a threshold value of *p* > .7 to assign individuals into categories (Figure 2, Table S4). All the reference samples were correctly assigned to the parental species (*p* > .997) and only F1 hybrids were identified (*p* > .780) from the putative hybrid population, the remainder of the individuals were identified as blesbok (*p* > .999) (Table 2; Table S4).

**TABLE 2:** List of samples indicating assignment of animals.

Sample ID	Age estimate	Sex	Genetic result/assignment	STRUCTURE		NEWHYBRIDS			
				Red hartebeest (*q*_i_)	Blesbok (*q*_i_)	Blesbok Jeffreys prior (*p*)	F1 Jeffreys prior (*p*)	Blesbok Jeffreys prior (*p*)	F1 uniform prior (*p*)
**1**	**Adult**	**Male**	**Hybrid**	**0.518**	**0.482**	**.0000**	**.9071**	**.0000**	**.9726**
**2**	**Adult**	**Male**	**Hybrid**	**0.562**	**0.438**	**.0000**	**1.0000**	**.0000**	**.9999**
3	Sub‐adult	Male	Blesbok	0.017	0.983	1.0000	.0000	1.0000	.0000
**4**	**Adult**	**Female**	**Hybrid**	**0.5988**	**0.4013**	**.0000**	**.9939**	**.0000**	**.9971**
5	Adult	Female	Blesbok	0.006	0.994	1.0000	.0000	1.0000	.0000
6	Adult	Male	Blesbok	0.002	0.998	1.0000	.0000	1.0000	.0000
7	Adult	Male	Blesbok	0.002	0.998	1.0000	.0000	1.0000	.0000
8	Adult	Female	Blesbok	0.002	0.998	1.0000	.0000	1.0000	.0000
9	Adult	Male	Blesbok	0.002	0.998	1.0000	.0000	1.0000	.0000
**10**	**Adult**	**Female**	**Hybrid**	**0.562**	**0.438**	**.0000**	**.9997**	**.0000**	**.9998**
11	Sub‐adult	Male	Blesbok	0.002	0.998	1.0000	.0000	1.0000	.0000
12	Adult	Male	Blesbok	0.002	0.998	1.0000	.0000	1.0000	.0000
**13**	**Adult**	**Male**	**Hybrid**	**0.6273**	**0.3727**	**.0000**	**.8358**	**.0000**	**.9742**
14	Adult	Female	Blesbok	0.002	0.998	1.0000	.0000	1.0000	.0000
**15**	**Adult**	**Female**	**Hybrid**	**0.5218**	**0.4783**	**.0000**	**1.0000**	**.0000**	**1.0000**
16	Adult	Female	Blesbok	0.002	0.998	1.0000	.0000	1.0000	.0000
17	Adult	Female	Blesbok	0.002	0.998	1.0000	.0000	1.0000	.0000
**18**	**Adult**	**Female**	**Hybrid**	**0.547**	**0.453**	**.0000**	**.7802**	**.0000**	**.9721**
19	Adult	Female	Blesbok	0.003	0.997	1.0000	.0000	.9999	.0000
20	Adult	Female	Blesbok	0.002	0.998	1.0000	.0000	1.0000	.0000
21	Adult	Female	Blesbok	0.002	0.998	1.0000	.0000	1.0000	.0000
22	Adult	Female	Blesbok	0.003	0.997	1.0000	.0000	.9999	.0000
23	Adult	Female	Blesbok	0.013	0.987	1.0000	.0000	.9999	.0000
24	Adult	Female	Blesbok	0.002	0.998	1.0000	.0000	1.0000	.0000
25	Adult	Female	Blesbok	0.002	0.998	1.0000	.0000	1.0000	.0000
26	Juvenile	Female	Blesbok	0.01	0.99	1.0000	.0000	.9999	.0000

*Note*: Identified hybrids are indicated in bold text.

**FIGURE 3:**
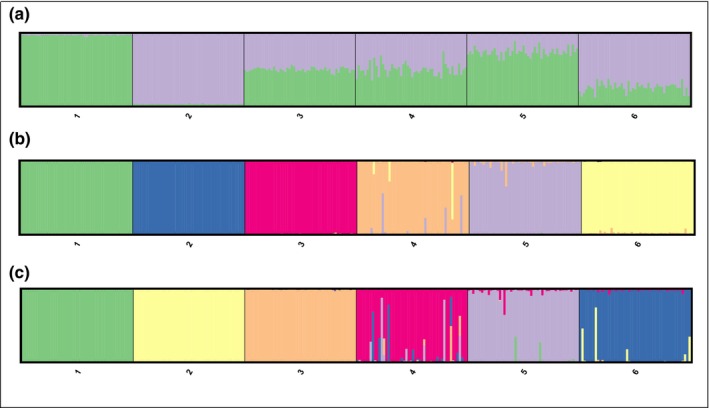
Proportional memberships of each genetic cluster and hybrid class for simulated individuals, estimated in (a) STRUCTURE performed with *K* = 2 and NEWHYBRIDS executed with (b) Jeffreys prior and (c) Uniform prior for 1 = Red hartebeest, 2 = Blesbok, 3 = F1 hybrids, 4 = F2 hybrids, 5 = backcross to red hartebeest and 6 = backcross to blesbok.

STRUCTURE identified all the simulated genotypes correctly except for one backcross to red hartebeest individual when applying a threshold value of *q*_i_ > 0.90 (Figure 3; Table S5). For NEWHYBRIDS the Jeffreys prior performed slightly better in assigning individuals to the correct category when using a threshold of *p* > .7 (Figure 3; Table S5). Analysing the simulated dataset using Jeffreys prior incorrectly categorised three F2 individual (Table S5) while applying the Uniform prior did not correctly identify eight F2 hybrids, two backcross to red hartebeest and three backcross to blesbok individuals (Table S5).

The average pairwise coefficient of relatedness in the putative hybrid group was −0.039. Within the seven F1 hybrids identified using STUCTURE and NEWHYBRIDS, first‐order relatives (0.48–0.58) and second‐order relatives (0.19–0.38) were identified indicating that they were either full‐siblings or half‐siblings. In all cases the parents could not be assigned indicating that they were culled prior to sampling.

## 4 DISCUSSION

Here, we describe the first application of genetic tools to identify hybrids, due to mating between a male red hartebeest and female blesbok. Hybridization is generally prevented due to pre‐zygotic barriers where behaviour such as courtship displays and chemical signals play a fundamental role in species‐specific mate recognition (Smadja & Butlin, 2009) and post‐zygotic barriers where hybrids are either sterile or have reduced fertility (Coyne & Orr, 2004; Dobzhansky, 1937; Mayr, 1963). In this study, hybridization was forced with the shortage of conspecific mates, specifically for the red hartebeest. Hybridization events due to changes in population demographics such as skewed sex ratios is well described in other species including Grevy's zebra (*Equus grevyi*) and plains zebra (*E. burchelli*; Cordingley et al., 2009), European mink (*Mustela lutreola*) and the polecat (*M. putorius*; Cabria et al., 2011) and fur seals (*Arctocephalus gazelle*, *A. tropicalis*, *A. forsteri*; Lancaster et al., 2006).

The seven hybrid individuals identified were considered sterile, mainly due to the presence of underdeveloped testes in the three hybrid males. In addition, Bayesian clustering analysis identified only F1 hybrids in the group further confirming sterility of the hybrids. In cases where hybrids are fertile, multigeneration hybrids and backcrosses would be expected. This finding is in line with a previous report of sterility in a red hartebeest x blesbok hybrid individual (Robinson et al., 1991). The species have different chromosome numbers which could lead to chromosome pairing complications during meiosis producing sterile offspring (Robinson et al., 1991). Experimental crossing of sibling species in mosquitos from the *Anopheles gambiae* complex has been reported to result in hybrid individuals with underdeveloped testes or hybrid individuals with normal testes morphologies and non‐motile spermatozoa depending on the direction of the crossings (Liang & Sharakhov, 2019). Anthropogenic hybridization with few or sterile offspring is characterised by wasted reproductive effort where there is no gene exchange between parental species (Allendorf et al., 2001). In these cases, consequences of concern are more ecological rather than genetic, such as slow population growth (Senanan et al., 2004). Hybridization resulting in wasted reproductive effort can lead to extinction if a threatened species is involved in hybrid mating with a more common species. An example is the bull trout (*Salvelinus confluentus*) that almost disappeared from a stream in Montana (USA) after the introduction of brook trout (*Salvelinus fontinalis*). The brook trout out‐numbered the bull trout which led to initiation of hybridization between the two species (Leary et al., 1993). The number of bull trout thus declined over time as the number of sterile offspring increased.

Anthropogenic hybridization occurs when there is removal of reproductive barriers between species that includes translocation of species outside their former distribution ranges leading to a breakdown in geographical isolation (Allendorf et al., 2001) and in addition the failure to ensure the presence of enough conspecific mates of the translocated animals (Robinson et al., 1991). The red hartebeest and blesbok is no different with both species having different habitat preferences that should prevent hybridization under normal conditions. The red hartebeest is more tolerant of woodland areas and high grass (IUCN SSC Antelope Specialist Group, 2017), while the blesbok prefers open grasslands (Dalton et al., 2017). Thus far, all recorded hybridization events between the two species are due to translocated animals not having conspecific mates (Robinson et al., 1991). Robinson et al. (1991) reported two different hybridization events, in both instances one red hartebeest male survived after translocation of a subpopulation which lead to hybridization with female blesbok. Thus, the long‐term negative consequences of hybridization between these two species may be limited. However, it is recommended that management plans should include details with regards to the availability of sufficient conspecific mates, where both species are managed sympatrically in order to reduce the risk of hybridization.

## AUTHOR CONTRIBUTIONS

**Anna M. van Wyk:** Formal analysis (equal); investigation (equal); methodology (equal); writing – original draft (equal). **Erika Schulze:** Investigation (equal); project administration (equal); writing – review and editing (equal). **Kim Labuschagne:** Methodology (equal); resources (equal); writing – review and editing (equal). **Seeng Thamae:** Methodology (equal); resources (equal); writing – review and editing (equal). **Antoinette Kotzé:** Writing – review and editing (equal). **Desiré Lee Dalton:** Conceptualization (equal); formal analysis (equal); methodology (equal); writing – review and editing (equal).

## CONFLICT OF INTEREST STATEMENT

The authors declare no conflicts of interest.

## Supporting information


Figure S1.

Tables S1–S5.

## Data Availability

All results have been deposited in Dryad (https://doi.org/10.5061/dryad.69p8cz98h).
