# Supplementary material for: Hybridization in an isolated population of blesbok and red hartebeest
Source: Ecol Evol. 2024 Apr 1;14(4):e11194. doi: 10.1002/ece3.11194 (PMC10985385; doi:10.1002/ece3.11194)
Supplement: Supplementary file 1 — Figure S1. [file ECE3-14-e11194-s002.docx]

| \|  \| 2008 \|  \| Founder population of 5 blesbok (gender unknown). \|  \| \| --- \| --- \| --- \| --- \| --- \| \|  \|  \|  \|  \|  \| \|  \| 2013 \|  \| One adult red hartebeest male and 19 blesbok added. Seven of the adult blesbok were culled. \|  \| \|  \|  \|  \|  \|  \| \|  \| 2014 \|  \| *Six offspring born* with two hybrid offspring suspected. Six blesbok were culled. \|  \| \|  \|  \|  \| . \|  \| \|  \| 2015 \|  \| *Five offspring born*. One blesbok mortality. \|  \| \|  \|  \|  \|  \|  \| \|  \| 2016 \|  \| *Five offspring born*. Two mortalities \|  \| \|  \|  \|  \|  \|  \| \|  \| 2017 \|  \| *Four offspring born*. Three mortalities. \|  \| \|  \|  \|  \|  \|  \| \|  \| 2018 \|  \| *Three offspring born*. To mortalities. Red hartebeest culled.  Collection of 26 tissue samples *post mortem.* \|  \| |
| --- | --- | --- | --- | --- | --- | --- | --- | --- | --- | --- | --- | --- | --- | --- | --- | --- | --- | --- | --- | --- | --- | --- | --- | --- | --- | --- | --- | --- | --- | --- | --- | --- | --- | --- | --- | --- | --- | --- | --- | --- | --- | --- | --- | --- | --- | --- | --- | --- | --- | --- | --- | --- | --- | --- | --- | --- | --- | --- | --- | --- | --- | --- | --- | --- | --- |
