# Supplementary material for: Hybridization in an isolated population of blesbok and red hartebeest
Source: Ecol Evol. 2024 Apr 1;14(4):e11194. doi: 10.1002/ece3.11194 (PMC10985385; doi:10.1002/ece3.11194)
Supplement: Supplementary file 2 — Tables S1–S5. [file ECE3-14-e11194-s001.zip › ece311194-sup-0002-TablesS1-S3.docx]

**Table S1**: Primer details and PCR conditions for amplification of microsatellite loci

| **Microsatellite marker** | **Primer sequence** | **Size range (bp)** | **Repeat unit** | **Fluorescent label** | **T_a_ (°C)** | **Reference** |
| --- | --- | --- | --- | --- | --- | --- |
| BM415 | F: 5’- gct aca gcc ctt ctg gtt tg -3’ | 131-171 | di-nucleotide | FAM | 50 | Bishop *et al*., 1994 |
| BM415 | R: 5’- gag cta atc acc aac agc aag -3’ |  |  |  |  |  |
| BM203 | F: 5’- ggg tgt gac att ttg ttc cc -3’ | 201-280 | di-nucleotide | FAM | 52 | Bishop *et al*., 1994 |
| BM203 | R: 5’- ctg ctc gcc act agt cct tc -3’ |  |  |  |  |  |
| ETH10 | F: 5’- gtt cag gac tgg ccc tgc taa ca -3’ | 200-230 | di-nucleotide | VIC | 62 | Toldo *et al*., 1993 |
| ETH10 | R: 5’- cct cca gcc cac ttt ctc ttc tc -3’ |  |  |  |  |  |
| OARFCB48 | F: 5’- gac tct aga gga tcg caa aga acc -3’ | 160-180 | di-nucleotide | PET | 55 | Buchanan *et al*., 1994 |
| OARFCB48 | R: 5’- gag tta gta caa gga tga caa gag gca c -3’ |  |  |  |  |  |
| BM2113 | F: 5’- gct gcc ttc tac caa ata ccc -3’ | 120-145 | di-nucleotide | VIC | 58 | Sunden *et al*., 1993 |
| BM2113 | R: 5’- ctt cct gag aga agc aac acc -3’ |  |  |  |  |  |
| OARFCB304 | F: 5’- ccc tag gag ctt tca ata aag aat cgg -3’ | 120-160 | di-nucleotide | NED | 55 | Buchanan et al., 1993 |
| OARFCB304 | R: 5’- cgc tgc tgt caa ctg ggt cag gg -3’ |  |  |  |  |  |
| OARCP26 | F: 5’- ggc cta aca gaa ttc aga tga tgt tgc -3’ | 110-170 | di-nucleotide | FAM | 58 | Ede *et al*., 1995 |
| OARCP26 | R: 5’- gtc acc ata ctg acg gct ggt tcc -3’ |  |  |  |  |  |
| BB03 | F: 5’- agc cat gtg cca atc ata tac t -3’ | 225-290 | Tri-nucleotide | FAM | 60 | Dalton et al., 2011 |
| BB03 | R: 5’- gga cac gga ctg aag cta ctt a -3’ |  |  |  |  |  |
| BB04 | F: 5’- ata aag gca tgt acc cca cat c -3’ | 140-160 | Tri-nucleotide | VIC | 62 | Dalton *et al*., 2011 |
| BB04 | R: 5’- cag aca gga ctg aag cga att a -3’ |  |  |  |  |  |
| BM1824 | F: 5’- gag caa ggt gtt ttt cca atc -3’ | 190-215 | di-nucleotide | FAM | 52 | Bishop *et al*., 1994 |
| BM1824 | R: 5’- cat tct cca act gct tcc ttg -3’ |  |  |  |  |  |
| BB05 | F: 5’- atg gac aga gga gcc tag tga g -3’ | 130-145 | di-nucleotide | PET | 60 | Dalton *et al*., 2011 |
| BB05 | R: 5’- act gtg cct ttc aac act gga -3’ |  |  |  |  |  |
| BB08 | F: 5’- acc tcc ctg tgg atg act tct -3’ | 165-180 | di-nucleotide | VIC | 60 | Dalton *et al*., 2011 |
| BB08 | R: 5’- gcc atg act gag caa cta aac a -3’ |  |  |  |  |  |
| BB10 | F: 5’- aat ggg gac aat gac gta cct a -3’ | 190-220 | di-nucleotide | NED | 58 | Dalton et al., 2011 |
| BB10 | R: 5’- aac agg aac cag ata gtg agt gg -3’ |  |  |  |  |  |
| BB20 | F: 5’- gct ctc cac ctt atg ctc atc t -3’ | 180-195 | di-nucleotide | VIC | 58 | Dalton et al., 2011 |
| BB20 | R: 5’- aac aca tgg cct gac tct ctt t -3’ |  |  |  |  |  |
| SRCRSP8 | F: 5’- tgc ggt ctg gtt ctg att tca c -3’ | 210-260 | di-nucleotide | NED | 50 | Bhebhe et al., 1994 |
| SRCRSP8 | R: 5’- gtt tct tcc tgc atg aga aag tcg atg ctt ag -3’ |  |  |  |  |  |
| TGLA263 | F: 5’- cga att cca aat ctg tta att tgc t -3’ | 120-170 | di-nucleotide | PET | 50 | Georges & Massey, 1992 |
| TGLA263 | R: 5’- aca gac aga aac tca atg aaa gca -3’ |  |  |  |  |  |
| INRA006 | F: 5’- agg aat atc tgt atc aac ctc agt c -3’ | 90-120 | di-nucleotide | FAM | 50 | Vaiman et al., 1992 |
| INRA006 | R: 5’- ctg agc tgg ggt ggg agc tat aaa ta -3’ |  |  |  |  |  |
| BMC3224 | F: 5’- cca tca ctg cta ttc tac ctc c -3’ | 210-240 | di-nucleotide | FAM | 55 | Bishop et al., 1994 |
| BMC3224 | R: 5’- cac agc caa ttt ctg att tca -3’ |  |  |  |  |  |
| SPS113 | F: 5’- cct cca caca gg ctt ctc tga ctt -3’ | 140-170 | di-nucleotide | FAM | 55 | FAO, 2011 |
| SPS113 | R: 5’- cct aac ttg ctt gag tta ttg ccc -3’ |  |  |  |  |  |
| INRA128 | F: 5’- taa gca ccg cac agc aga tgc -3’ | 160-210 | di-nucleotide | NED | 55 | Vaiman et al., 1994 |
| INRA128 | R: 5’- aga cta gtc agg ctt cct ac -3’ |  |  |  |  |  |
| BMS4008 | F: 5’- cgg ccc taa gtg ata tgt tg -3’ | 140-180 | di-nucleotide | VIC | 55 | Sonstegard et al., 1997 |
| BMS4008 | R: 5’- gaa gag tgt gag gga aag act g -3’ |  |  |  |  |  |

Polymerase Chain Reaction (PCR) amplification was conducted in a 12.5 micro litre (µL) reaction volume consisting of AmpliTaq^®^ DNA polymerase (Roche Molecular Systems, Inc), forward and reverse primers (0.5 micro Molar (µM) each) and 50 nano gram (ng) genomic DNA template. The conditions for PCR amplification were as follows: 5 minutes (min) at 95°C denaturation, 35 cycles for 30 seconds (sec) at 95°C, 30 sec at 50 – 62°C (supplementary Table 1) and 30 sec at 72°C, followed by extension at 72°C for 10 min in a T100™ Thermal Cycler (Bio-Rad Laboratories, Inc. Hercules, CA, USA). PCR products were run against a Genescan™ 500 LIZ™ internal size standard on an ABI 3130 genetic analyser (Applied Biosystems, Inc., Foster City, CA, USA) and were genotyped using GeneMapper® v. 4.0 (Applied Biosystems, Inc., Foster City, CA, USA).

**Table S2**: Null allele estimation for the red hartebeest and blesbok reference populations calculated using four statistical methodologies (Oosterhout, Chakraborty, Brookfield 1 and Brookfield 2) in MICRO-CHECKER. Presence of null alleles is indicated (yes) if the average null allele frequencies are above 0.2 at all four algorithms.

| **Red hartebeest population** | | | | | |
| --- | --- | --- | --- | --- | --- |
| Locus | Null present | Oosterhout | Chakraborty | Brookfield 1 | Brookfield 2 |
| OARFCB48 | no | 0.0084 | 0.0069 | 0.0064 | 0.0064 |
| OARFCB304 | no | 0.0268 | 0.0226 | 0.0197 | 0.0197 |
| BM415 | no | 0.1544 | 0.2571 | 0.0845 | 0.0845 |
| TGLA263 | no | 0.0594 | 0.0545 | 0.0451 | 0.0451 |
| INRA006 | no | -0.0305 | -0.0306 | -0.028 | 0 |
| BB03 | no | 0 | 0 | 0 | 0 |
| BB08 | no | 0.0565 | 0.0471 | 0.0381 | 0.1965 |
| OARCP26 | no | -0.1229 | -0.0612 | -0.0221 | 0 |
| BB10 | no | 0.1205 | 0.106 | 0.0697 | 0.0697 |
| BM2113 | no | -0.1229 | -0.0612 | -0.0221 | 0 |
| BB20 | no | 0 | 0 | 0 | 0 |
| BB04 | no | -0.1229 | -0.0612 | -0.0221 | 0 |
| ETH10 | no | -0.0177 | 0.0038 | 0.0033 | 0.0033 |
| BM1824 | no | 0.0657 | 0.028 | 0.0179 | 0.0179 |
| BM203 | no | -0.0267 | -0.028 | -0.0269 | 0 |
| BMC3224 | no | -0.0385 | -0.0256 | -0.0222 | 0 |
| BMS4008 | no | 0.1274 | 0.151 | 0.1194 | 0.1194 |
| INRA128 | no | -0.0534 | -0.0269 | -0.0246 | 0 |
| **Blesbok population** | | | | | |
| Locus | Null Present | Oosterhout | Chakraborty | Brookfield 1 | Brookfield 2 |
| OARFCB48 | no | -0.1728 | -0.0857 | -0.0394 | 0.1687 |
| OARFCB304 | no | 0.0056 | -0.0077 | -0.006 | 0.1392 |
| BM415 | no | 0.2245 | 0.392 | 0.1495 | 0.2719 |
| TGLA263 | no | 0.0599 | 0.072 | 0.0508 | 0.1785 |
| INRA006 | no | 0.1838 | 0.2024 | 0.1202 | 0.2342 |
| BB03 | no | -0.2828 | -0.127 | -0.0812 | 0.2153 |
| BB08 | yes | 0.2126 | 0.286 | 0.1668 | 0.3397 |
| OARCP26 | yes | 0.203 | 0.3287 | 0.1511 | 0.3437 |
| BB10 | yes | 0.2937 | 0.5232 | 0.2387 | 0.3977 |
| BM2113 | yes | 0.2352 | 0.3833 | 0.1843 | 0.3612 |
| BB20 | no | -0.0121 | -0.0272 | -0.0205 | 0.215 |
| BB04 | yes | 0.2724 | 0.4693 | 0.2017 | 0.3765 |
| ETH10 | yes | 0.2331 | 0.3246 | 0.1728 | 0.349 |
| BM1824 | no | 0.1828 | 0.3112 | 0.1097 | 0.2499 |
| BM203 | no | 0.1921 | 0.2963 | 0.1277 | 0.2542 |
| BMC3224 | no | 0.0202 | 0.0478 | 0.0355 | 0.1645 |
| BMS4008 | no | -0.0261 | -0.0256 | -0.0175 | 0.1489 |
| INRA128 | yes | 0.2138 | 0.3174 | 0.1824 | 0.2789 |

**Table S3**: Allele frequencies for all loci in red hartebeest and blesbok populations with private allele frequencies indicated in bold.

| **Locus** | **Allele** | **Red hartebeest** | **Blesbok** |
| --- | --- | --- | --- |
| **OARFCB48** | | | |
|  | 149 | **0,038** | 0,000 |
|  | 151 | **0,192** | 0,000 |
|  | 153 | **0,077** | 0,000 |
|  | 161 | **0,077** | 0,000 |
|  | 163 | **0,192** | 0,000 |
|  | 165 | 0,115 | 0,825 |
|  | 167 | 0,038 | 0,175 |
|  | 169 | **0,192** | 0,000 |
|  | 173 | **0,038** | 0,000 |
|  | 179 | **0,038** | 0,000 |
| **OARFCB304** | | | |
|  | 133 | 0,000 | **0,300** |
|  | 135 | 0,000 | **0,200** |
|  | 139 | 0,000 | **0,500** |
|  | 141 | **0,115** | 0,000 |
|  | 143 | **0,346** | 0,000 |
|  | 145 | **0,154** | 0,000 |
|  | 149 | **0,038** | 0,000 |
|  | 151 | **0,077** | 0,000 |
|  | 153 | **0,038** | 0,000 |
|  | 155 | **0,077** | 0,000 |
|  | 157 | **0,154** | 0,000 |
| **BM415** | | | |
|  | 145 | 0,846 | 0,225 |
|  | 147 | **0,154** | 0,000 |
|  | 153 | 0,000 | **0,775** |
| **TGLA263** | | | |
|  | 95 | **0,115** | 0,000 |
|  | 105 | **0,115** | 0,000 |
|  | 107 | **0,385** | 0,000 |
|  | 109 | **0,115** | 0,000 |
|  | 111 | **0,038** | 0,000 |
|  | 115 | **0,192** | 0,000 |
|  | 131 | 0,000 | **0,500** |
|  | 137 | 0,000 | **0,375** |
|  | 139 | 0,038 | 0,100 |
|  | 149 | 0,000 | **0,025** |
| **INRA006** | | | |
|  | 98 | 0,000 | **0,625** |
|  | 100 | **0,038** | 0,000 |
|  | 104 | **0,038** | 0,000 |
|  | 106 | 0,000 | **0,175** |
|  | 108 | **0,115** | 0,000 |
|  | 114 | 0,000 | **0,200** |
|  | 116 | **0,115** | 0,000 |
|  | 118 | **0,077** | 0,000 |
|  | 120 | **0,269** | 0,000 |
|  | 122 | **0,308** | 0,000 |
|  | 124 | **0,038** | 0,000 |
| **BB03** | | | |
|  | 232 | **1,000** | 0,000 |
|  | 244 | 0,000 | **0,026** |
|  | 264 | 0,000 | **0,211** |
|  | 267 | 0,000 | **0,763** |
| **BB20** | | | |
|  | 185 | **1,000** | 0,000 |
|  | 187 | 0,000 | **0,579** |
|  | 189 | 0,000 | **0,289** |
|  | 191 | 0,000 | **0,132** |
| **BM1824** | | | |
|  | 188 | **0,077** | 0,000 |
|  | 192 | **0,692** | 0,000 |
|  | 194 | **0,038** | 0,000 |
|  | 196 | **0,038** | 0,000 |
|  | 198 | 0,000 | **0,825** |
|  | 200 | 0,000 | **0,175** |
|  | 202 | **0,154** | 0,000 |
| **BM203** | | | |
|  | 224 | 0,000 | **0,750** |
|  | 228 | 0,000 | **0,250** |
|  | 238 | **0,038** | 0,000 |
|  | 244 | **0,115** | 0,000 |
|  | 252 | **0,231** | 0,000 |
|  | 254 | **0,115** | 0,000 |
|  | 256 | **0,154** | 0,000 |
|  | 258 | **0,038** | 0,000 |
|  | 260 | **0,038** | 0,000 |
|  | 262 | **0,077** | 0,000 |
|  | 266 | **0,077** | 0,000 |
|  | 272 | **0,077** | 0,000 |
|  | 276 | **0,038** | 0,000 |
| **BMC3224** | | | |
|  | 213 | **0,231** | 0,000 |
|  | 215 | **0,038** | 0,000 |
|  | 219 | **0,385** | 0,000 |
|  | 221 | **0,115** | 0,000 |
|  | 223 | 0,231 | 0,025 |
|  | 227 | 0,000 | **0,525** |
|  | 231 | 0,000 | **0,225** |
|  | 233 | 0,000 | **0,225** |
| **BMS4008** | | | |
|  | 142 | **0,038** | 0,000 |
|  | 144 | **0,077** | 0,000 |
|  | 158 | 0,192 | 0,650 |
|  | 162 | **0,192** | 0,000 |
|  | 164 | **0,038** | 0,000 |
|  | 166 | 0,077 | 0,250 |
|  | 168 | 0,038 | 0,100 |
|  | 170 | **0,038** | 0,000 |
|  | 172 | **0,269** | 0,000 |
|  | 180 | **0,038** | 0,000 |
